# BCG mediated protection of the lung against experimental SARS-CoV-2 infection

**DOI:** 10.3389/fimmu.2023.1232764

**Published:** 2023-09-08

**Authors:** Kerry L. Hilligan, Sivaranjani Namasivayam, Alan Sher

**Affiliations:** ^1^ Immunobiology Section, Laboratory of Parasitic Diseases, National Institute of Allergy and Infectious Diseases, Bethesda, MD, United States; ^2^ Immune Cell Biology Programme, Malaghan Institute of Medical Research, Wellington, New Zealand

**Keywords:** COVID-19, Bacille Calmette-Guérin (BCG), lung, interferon gamma (IFNγ), trained immunity

## Abstract

The observation of reduced COVID-19 incidence and severity in populations receiving neonatal intradermal BCG vaccination vaccine raised the question of whether BCG can induce non-specific protection against the SARS-CoV-2 (SCV2) virus. Subsequent epidemiologic studies and clinical trials have largely failed to support this hypothesis. Furthermore, in small animal model studies all investigators have failed to observe resistance to viral challenge in response to BCG immunization by the conventional and clinically acceptable intradermal or subcutaneous routes. Nevertheless, BCG administered by the intravenous (IV) route has been shown to strongly protect both hamsters and mice against SCV2 infection and disease. In this Perspective, we review the current data on the effects of BCG vaccination on resistance to COVID-19 as well as summarize recent work in rodent models on the mechanisms by which IV administered BCG promotes resistance to the virus and discuss the translational implications of these findings.

## Introduction

The innate compartment of the immune system differs from the adaptive in its ability to provide non-specific defense against a wide variety of threats encountered by the body and its stimulation is an important strategy for enhancing host resistance to pathogens. Avirulent microbes and their products are themselves important triggers of innate immune function and recently have been described to do so with long term effects (1). BCG (Bacille Calmette Guérin) is a well-studied microbial stimulus for its effects on innate immunity. This attenuated *Mycobacterium bovis* strain is widely used to vaccinate against extrapulmonary tuberculosis (TB) in infants and children and was discovered in the mid-20^th^ century to also promote non-specific resistance against tumors, a finding that led to its current employment as a treatment for some forms of bladder cancer (2). More recently BCG vaccination has been associated with lowering all-cause mortality in infants (3), reducing viremia after a yellow fever vaccine challenge in adults (4), and decreasing risk of respiratory infections in the elderly (5). Multiple mechanisms have been proposed to explain these effects, the most prominent of which involve the induction of “trained immunity” in which myelopoietic-derived innate effector cells become epigenetically modified so that they remain in a long-term primed state (up to 1 year in humans) (6–8).

BCG is typically administered to vaccinees by intradermal (ID) or subcutaneous (SC) injection although other routes (e.g. oral) have been employed in the past (9, 10). Although not clinically approved, the intravenous (IV) route of BCG administration has recently been employed in two important studies related to TB vaccination. In the first study, Kaufmann and colleagues showed that IV BCG preferentially induces trained immunity in mice because of its ability to access and infect long lived myelopoietic stem cells in the bone marrow (7). In the second report, Darrah and colleagues showed that IV in contrast to SC administered BCG induces sterile immunity against *M. tuberculosis* (Mtb) challenge in a rhesus monkey model (11), a dramatic finding that the authors attributed to the direct targeting of the lung and the induction of a strong local memory T cell response when the vaccine is given by this route (12). Recent studies indicate that in macaques such resistance can persist after the clearance of culturable BCG bacilli (13). Nevertheless, the contribution of BCG stimulated innate immune mechanisms to this striking protection is at present unclear.

## Clinical evidence for or against the association of BCG vaccination with host resistance to COVID-19

Given its previously demonstrated ability to stimulate non-specific host resistance to certain other viral infections, BCG immunization was suggested in the early months of the COVID-19 pandemic as a possible prophylactic measure for the prevention of SCV2 infection and disease (14, 15). This concept was initially supported by a number of ecological/epidemiologic studies suggesting an association of prior BCG vaccination with a lower incidence of COVID-19 disease (16, 17) despite the relatively short period (up to 1 year) that “trained” responses have been reported to persist *in vivo* (8). This early work was followed up with a large number of more extensive investigations (summarized in **Table 1**) that in general have failed to confirm the protective effects of BCG vaccination on the incidence and severity of SCV2 infection (18–21, 26–29), including a recently published international multi-cohort randomized trial (BRACE) involving ID administration of BCG to adult health care workers (22). One study conducted with a small cohort of older adults in Greece did note some protection against the incidence of COVID-19 symptoms; however, the existence of SCV2 infection in these individuals was not confirmed by PCR or antibody testing (24). A significant reduction in the incidence and symptom severity of COVID-19 was also observed in a different study involving the follow-up of adult diabetes patients given 3 ID doses of intramural BCG over a 2-3 year period before the onset of the pandemic (25). The explanation for the unusual efficacy observed in the latter study is unclear but may relate to the multiple dosage, the use of a highly virulent BCG isolate (Tokyo strain) (30, 31), the spacing between BCG vaccination and SCV2 exposure, or possibly the diabetic state of the participants. Overall, there is currently no compelling evidence that a single-dose intradermal BCG inoculation provides protection against SCV2 infection and disease; however, there may be certain conditions that favor the protective outcomes observed with multiple BCG doses (25). Future studies examining prolonged or repeated mycobacterial exposures, either due to population level exposure to environmental mycobacteria and/or BCG re-vaccination strategies, may provide further insights into any potential protective effects (32).

**Table 1:** Summary of human trials investigating BCG efficacy against COVID-19.

Trial design	Participant characteristics	BCG strain	SCV2 outcomes (versus control arm)			Reference
			Incidence	Severity	Other parameters	
RCT, ~1000 individuals/arm	Adult, 60y+	Danish 1331	NC	NA	Higher SCV2 antibody titers in BCG vaccinated participants	(18)
RCT, ~1000 individuals/arm	Adult, 60y+	VPM1002	NC	NA	NC in self-reported duration of illness with respiratory tract infection, but trend towards lower duration in BCG vaccinated individuals within the cohort who did not received COVID-19-specific vaccines.	(19)
RCT, ~750 individuals/arm	Adult, health care workers	Danish 1331	NC	NC		(20, 21)
RCT, ~1700 individuals/arm	Adult, health care workers	Danish 1331	NC	NC	Lower cytokine responses in whole blood samples exposed to irradiated SCV2 in BCG vaccinated individuals (n=25)	(22, 23)
RCT, ~150 individuals/arm	Adult, 50y+	Moscow	Reduced*	NA		(24)
RCT, 48 in placebo arm, 96 in BCG arm	Adult, type-1 diabetes patients	Tokyo 172, 3 doses	Reduced	Reduced		(25)
RCT, ~3000 individuals/arm	Adult, 60y+ with >1 co-morbidities	Danish 1331	NC	NC	NC in incidence of other respiratory infections	(26)
RCT, ~130 individuals/arm	Adult, health care workers	Moscow or Moreau	NC	NA		(27)
RCT, ~70 individuals/arm	Adult, health care workers	Moscow	NC	NA		(28)
RCT, ~250 individuals/arm	Adults	Not specified	NC	Reduced		(29)

NC, no change; NA, not assessed.

*COVID-19 incidence was defined as “possible/probable/definitive” in this study.

These citations are based on a literature search in May 2023.

## Evidence in animal models for BCG induced protection against SARS-CoV-2

The hypothesis that prior BCG vaccination might offer protection against COVID-19 prompted a series of studies in different animal models to examine the effects of prior BCG administration on resistance to SCV2 challenge (**Table 2**). This work has generated a consensus that when inoculated by the conventional ID (or subcutaneous) route to mice (33, 35–37) or hamsters (35, 38) or by aerosol to monkeys (39), BCG fails to trigger significant protection against intranasal or intra-bronchial infection with the virus. Nevertheless, a number of independent studies have shown that when administered by the IV route to mice or hamsters, BCG can confer high levels of resistance to both SCV2 infection and disease (**Table 2**) (33, 34, 38, 40, 41). In the initial description of this effect, K18 transgenic mice which express the human ACE2 receptor (K18-hACE2) for the virus were IV inoculated with BCG (Pasteur strain) before intranasal SCV2 infection with a lethal dose of the WA/2020 strain (33). At 42 days following BCG administration, the virus challenged mice showed a striking protection from SCV2 induced weight loss and mortality along with pronounced reductions in pulmonary viral loads at 5 days post infection. This protection was still evident 112 days following BCG inoculation but at lower levels (33). To confirm that the COVID-19 resistance induced by IV BCG is not peculiar to hACE2 transgenic mice, the experiments were repeated using a second model in which wild type C57BL/6 mice were challenged with the more virulent B.1.1.7 SCV2 variant. In this situation unvaccinated mice support viral replication for 3-4 days before clearing the infection with minimal accompanying disease. Again, IV BCG induced striking protection against SCV2 with the majority of the BCG exposed mice showing no detectable virus in their lungs at 3 days following B.1.1.7 challenge (33). Consistent with the other studies cited above, no significant resistance against SCV2 was observed in mice inoculated with the same dose of BCG by the SC route in either of the two murine models. The ability of IV BCG to protect K18-hACE2 mice from early SCV2 infection was confirmed in a second study using the Tokyo strain of BCG and intranasal viral challenge with either an original “wild-type” strain or more virulent kappa or delta variants (40). In additional work, IV administered BCG (Tice strain) was shown to reduce viral loads and bronchopneumonia in Syrian hamsters challenged intranasally with the Wuhan-1 strain SCV2 (38). In contrast to the above findings, Kaufmann et al. reported that K18-hACE2 mice or hamsters given IV (or SC) BCG (Tice strain) showed no significant protection against intranasal (or in the case of mice either intranasal or intratracheal) challenge with a SCV2-B lineage variant. Nevertheless, the same BCG exposed mice displayed resistance to intranasally administered Influenza A virus (35). Ongoing follow up studies suggest that the negative results with SCV2 obtained in the latter study may relate to the BCG strain (42), its preparation and/or the dose employed for vaccination (Kaufmann and Hilligan, unpublished).

**Table 2:** Summary of animal studies assessing efficacy of BCG against SCV2 infection and disease.

Animal model	Route of BCG administration	BCG strain	SCV2 outcomes (versus control group)		Reference
			Disease phenotype	Viral titers	
Mouse, K18-hACE2	SC	Pasteur	NC (survival and weight loss)	NC	(33)
Mouse, K18-hACE2	IV	Pasteur	Improved (survival and weight loss)	Reduced	(33)
Mouse, wildtype B6	SC	Pasteur	n/a	NC	(33)
Mouse, wildtype B6	IV	Pasteur	n/a	Reduced	(33, 34)
Mouse, K18-hACE2	SC	Tice	NC (survival and weight loss)	NC	(35)
Mouse, K18-hACE2	IV	Tice	NC (survival and weight loss)	NC	(35)
Hamster, Syrian Golden	SC	Tice	NC (weight loss)	NC	(35)
Hamster, Syrian Golden	IV	Tice	NC (weight loss)	NC	(35)
Hamster, Roborovski	SC	Tice	NC (survival and weight loss)	NC	(35)
Hamster, Roborovski	IV	Tice	NC (survival and weight loss)	NC	(35)
Mouse, K18-hACE2	SC	Pasteur	NC (weight loss)	NC	(36)
Mouse, K18-hACE2	SC	Pasteur	NC (survival and weight loss)	NC	(37)
Hamster, Syrian Golden	IV	Tice	Improved (bronchopneumonia score)	Reduced	(38)
Rhesus macaque	aerosol	Danish 1331	NC (pathology score)	NC	(39)
Mouse, K18-hACE2	SC	Tokyo 172	NA	NC	(40)
Mouse, K18-hACE2	IV	Tokyo 172	Modestly improved (weight loss)	Reduced	(40)
Mouse, wildtype B6	IV	Tice	Improved (weight loss)*	Reduced*	(41)

NC, no change; NA, not assessed; n/a, not applicable.

*protective effect only apparent from 21 days after IV BCG inoculation.

The consistent failure of SC or ID inoculated BCG to provide protection against SCV2 infection suggests that the resistance conferred by IV BCG may relate to the long-term presence mycobacteria in the lungs and accompanying granulomatous inflammation occurring in animals inoculated by that route (33, 40). Consistent with this hypothesis, K18-hACE2 or non-transgenic mice infected by aerosol with virulent *Mycobacterium tuberculosis* and developing pulmonary TB, display high levels of resistance to SCV2 comparable to that reported in IV BCG exposed animals (37, 43, 44). Nevertheless, as noted above, in rhesus macaques BCG given by the aerosol route failed to induce protection against SCV2 challenge (39). Since pulmonary bacterial infection and local tissue responses were not evaluated in that study, it is difficult to ascertain whether this discrepancy with the rodent studies reflects the different host species employed or the local levels of BCG and/or immune responses occurring at that site. Indeed, a comparison between IV and aerosol inoculation of rhesus macaques by Darrah et al, showed that only IV BCG resulted in the formation of “microgranuloma” structures in the lung as well as increased numbers of CD4+ T cells and CD11c+ antigen-presenting cells (11).

## Mechanisms underlying BCG induced resistance to SCV2 infection and disease

It was originally proposed that ID (or SC) administered BCG might offer protection against COVID-19 because of its previously documented ability to enhance clinical resistance to other viral infections, effects that were attributed to the induction of trained immunity (14, 15). Since in nearly all studies humans vaccinated with BCG by this route fail to display significant resistance to COVID-19, it would appear that any response induced by a single-dose BCG inoculation is not sufficient to restrict SCV2. Nevertheless, it is still possible that boosting of the response by intradermal re-vaccination could induce more effective immunity and this could be the basis of the protection against COVID-19 observed by Faustman and colleagues in diabetes patients given multiple BCG inoculations (25).

Since with the latter exception BCG induced protection against SCV2 has not been documented in humans or non-human primates, nearly all the current information on anti-viral mechanisms derives from the studies on murine and hamster rodents involving IV administered bacteria. That route of inoculation has been previously shown in mice to preferentially stimulate myelopoiesis and the generation of monocyte/macrophages with a trained phenotype (7). Consistent with these earlier findings, Zhang and colleagues reported that IV BCG vaccinated mice challenged with SCV2 display enhanced bone marrow myelopoiesis, augmented pulmonary monocyte/macrophage infiltration and upregulated innate immune and metabolic gene signatures previously described as associated with training (40). Although not specifically addressing the issue of trained immunity, both the NIH murine model study of Hilligan et al. and hamster study of Singh and colleagues described enhanced pulmonary macrophage numbers in IV BCG inoculated animals that likely arise from bone marrow monocytes (33, 38). Given the long-term persistence of both mycobacteria and granulomatous inflammation in the lungs of IV BCG vaccinated mice (33, 40), it is unlikely that resistance to SCV2 challenge would require the type of trained myeloid cells previously described as arising in hosts exposed to a prior single intradermal bacterial inoculation.

In each of the three studies documenting protection against SCV2 induced by IV BCG, vaccination was shown to simultaneously reduce pulmonary viral load and virus induced bronchopneumonia, in some cases as early as 2 days post challenge. Consistent with the latter observation, in both mouse studies BCG inoculation resulted in lowered production of SCV2 induced IL-6 and MCP1 (CCL2) (33, 40). Although this decrease could reflect an effect of reduced viral load in the vaccinated animals, the results of a multivariate analysis performed in the NIH murine study revealed an inhibitory effect of prior IV BCG administration on the induction of these pathology associated cytokines independent of viral titer (33). These data align with results from the BRACE clinical trial that showed that while BCG vaccination did not protect against COVID-19 (22), BCG did limit SCV2-induced pro-inflammatory cytokine responses *ex vivo*, suggesting that BCG inoculation can modulate virus triggered immune responses independent of its protective effect (23).

In both mice and hamsters, IV BCG administration led to pronounced elevations in pulmonary T cells, while only a minor response was seen in mice given SC BCG. In mice, IV BCG enhanced lymphocytes were characterized as CD8+, FoxP3− CD4+, and FoxP3+ CD4+ T cells, as well as MAIT cells and their levels did not significantly increase following viral challenge (33, 38). Indeed, if anything, prior IV BCG administration appeared to suppress the CD8+ T cell expansion triggered by SCV2 infection. Somewhat in contrast, in the hamster model, prior IV BCG inoculation resulted in an expansion of cells with Th1, Th17, Treg, CTLs or Tmem transcriptional markers after viral challenge as well as the emergence of a new plasma cell population not present prior to SCV2 exposure and expressing genes associated with immunoglobulin production suggestive of accelerated antibody production. In the same hamster study, IV BCG vaccination also appeared to dampen the expression of T cell exhaustion markers triggered by SCV2 infection (38). Together these observations show that IV BCG triggers the recruitment of adaptive immune cells into the lung tissue that in addition to supplying a potential source of protective antibodies may be important in providing cytokines and other signals that shape the innate immune landscape. Another interesting possibility is that the response to the bacteria has hindered the ability of the host to respond to another inflammatory stimuli.

**Figure 1:**
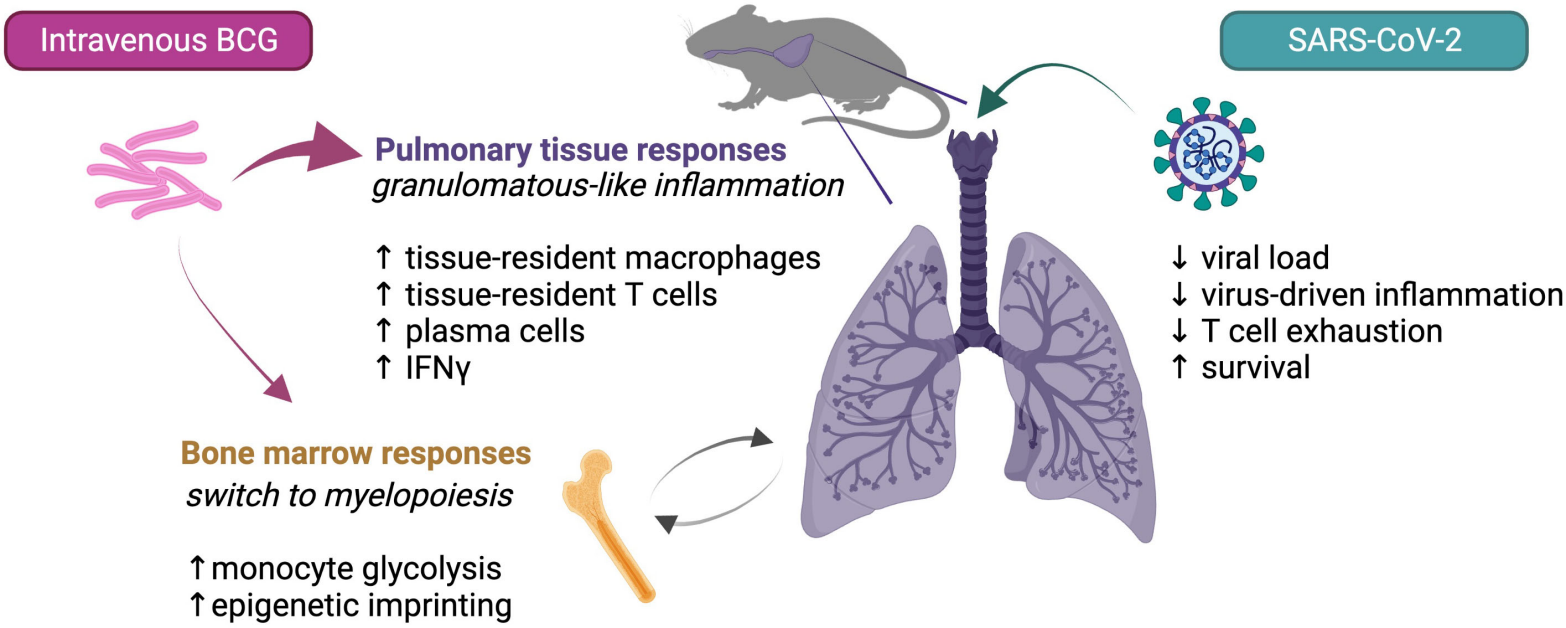
Possible mechanisms contributing to IV BCG conferred protection against SCV2 in mice.

Type I IFNs are important for control of viral pathogens but in SCV2 and other virus infections these cytokines can also promote pathology (45, 46). Interestingly in the NIH mouse model study prior IV BCG inoculation appeared to suppress rather than augment the SCV2 triggered Type I IFN response consistent with the suppression of COVID-19-like pathology observed in these animals. BCG infection is classically associated with strong IFNγ production from CD4+ T, CD8+ T and NK cells and the cytokine was found to be heavily induced in the lungs of both mice and hamsters months after IV BCG inoculation (33, 38, 40). Importantly, this local Type II IFN response was minimal in mice vaccinated by the SC route consistent with the dearth of both BCG and its associated granulomatous tissue inflammation in lungs of these animals in contrast to IV inoculated mice. Recent functional studies in the murine models suggest that this IFNγ response deriving primarily from CD4+ T cells and acting on non-hematopoietic cells in the lung is required for the reduction in both SCV2 virus and its associated pathology and that the recombinant cytokine itself can trigger these effects (34, 41). Whether IV BCG induced protection against SCV2 is mediated entirely through this mechanism or also involves the myeloid, T or B lymphocytes changes reported to be associated with resistance in the studies discussed above is at present unclear. A summary of the different effector mechanisms currently proposed to explain the protection against SCV2 induced by IV BCG is presented in **Figure 1**.

## Discussion and translational implications

The findings reviewed above establish a proof of principle in animal models that single dose BCG can stimulate protection against SCV2 but only when given IV, a mode of administration that is currently not clinically acceptable. The data do not rule out the possibility that through repeated boosting (25) or the use of a specially engineered bacterial strain (36) protection against COVID-19 could be generated through conventional ID or SC vaccination although it is likely that such resistance would involve a different mechanism. There is currently considerable interest in the possible use of IV administered BCG for vaccination against *M. tuberculosis* because of its ability to confer sterile immunity against this important pathogen in rhesus monkeys (11, 13). This has stimulated efforts to develop attenuated BCG mutants (e.g. auxotrophs) that would be safe for human intravenous use and such strains could be tested as candidates for protection against COVID-19 (47).

Regardless, the demonstration that bacterial stimulation of the lung can induce high levels of resistance against SCV2 could lead to the discovery of novel mechanisms of anti-viral protection with potential clinical applicability. For example, the recent evidence that BCG induced IFNγ can protect mice from SCV2 challenge (34, 41) raises the question of whether the cytokine could be used intranasally to protect subjects at high risk of infection possibly with less risk of toxicity than Type I IFN. It is also becoming clear that IV BCG is not a unique non-specific stimulus for host protection against experimental SCV2. In addition to prior *M. tuberculosis* infection (37, 43, 44), recent findings indicate that intranasally administered PRR ligands can also trigger host resistance in the same murine models (48–50) as can prior infection with a lung-transiting helminth (51). While seemingly distinct stimuli, it is possible that they all act by triggering the production of anti-viral effectors by pulmonary myeloid or epithelial cells.

As noted in the studies reviewed here, IV BCG infection can trigger long term changes in the cellular composition and adaptive immune responsiveness of lung tissue. While trained immunity may contribute [recently reviewed by Netea et al. (52)], other factors such as bacterial induced tissue remodeling and continuous immune stimulation by the bacteria surviving within granuloma-like structures in the lung are in this situation likely to play a more important role in promoting the long-lived property of the protection triggered by IV BCG at that tissue site (**Figure 1**).

Despite its limitations as a vaccine, studies on BCG continue to provide important insights on the interplay of innate and adaptive immunity in the host response to pathogens and in this case hopefully add to our understanding of how the lung can be stimulated to control both SCV2 and COVID-19 associated pathology.

## Author contributions

Writing-draft: KH, AS. Writing-review and editing: KH, SN, AS. All authors contributed to the article and approved the submitted version.
